# Wood’s Medical and Surgical Monographs

**Published:** 1890-04

**Authors:** 


**Wood's Medical and Surgical Monographs.** Published monthly. Vol. V., No. 1, January, 1890. Vol. V., No. 2, February, 1890. Vol. V., No. 3, March, 1890. Price, $10.00 a year; single copies, $1.00. New York: William Wood & Co. 1890.

I. The series for 1890 begins with an article on Neuralgia: Its Etiology, Diagnosis, and Treatment, by W. R. Gowers, M. D., F. R. C. S., which, though short, is full of interest, and should be read by every general practitioner. The other titles in this number are The Prognosis of Diseases of the Heart, by Prof. E. Leyden, of Berlin; The Sputum: A Contribution to Clinical Diagnosis and Practical Examination for Tubercle Bacilli, by Peter Kaatzer, M. D.; Hypnotism: Its Significance and Management briefly presented, by Dr. August Forel; and The Forms of Nasal Obstruction in Relation to Throat and Ear Diseases, by Greville Macdonald, M. D. This latter article takes up sixty-four pages of the book, and is of special interest to the larynogologist and otologist. This seems a convenient place for us to remark that there would appear no good reason why the laryngologist should not also practice otology, for the two branches are in close anatomical and sympathetic relations, whereas the ear has no such relation to the eye.

II. The February issue is made up as follows: The Formation and Excretion of Uric Acid, as Elucidating its Action in the Causation of Disease, by A. Haig, M. A., M. D.; The Initial Stages of Consumption: Their Nature and Treatment, Including Dietetic Suggestions, by Horace Dobell, M. D.; Ectopic Pregnancy and Pelvic Hematocele, by Lawson Tait, F. R. C. S., Edin. and Eng., etc. The chief interest in this number centers in Mr. Tait's masterly elucidation of one of the most important questions of the day, and the publishers have been most wise in their placing it within the reach of the entire profession. In the March number of this JOURNAL for 1889, (Vol. XXVIII., p. 462,) we reviewed this subject *in extenso* from the author's English edition, of which this monograph is a reprint, and to that we refer the reader for much of interest in connection with the subject of ectopic gestation.

III. The March number contains the following monographs: Treatment of Cancer by Electricity, by J. Inglis Parsons, M. D.; The Dreadful Revival of Leprosy, by Sir Morell Mackenzie, M. D.; Disease of Old Age, by Dr. A. Seidel; Urinary Neuroses of Childhood, by Dr. Louis J. Guinou; Varicose Veins of the Lower Extremities, by William H. Bennett, F. R. C. S.; The Uses of Electrolysis in Surgery, by W. E. Stevenson, M. D. Numerous illustrations, and an index, make Volume V. a fit companion to its predecessors.

